# Failed Spinal Anesthesia: Incidence and Associated Factors

**DOI:** 10.7759/cureus.76078

**Published:** 2024-12-20

**Authors:** Aparna Bagle, Shweta Khatri, Runjhun Jain

**Affiliations:** 1 Department of Anaesthesiology, Dr. D. Y. Patil Medical College, Hospital & Research Centre, Dr. D. Y. Patil Vidyapeeth (Deemed to be University), Pune, IND

**Keywords:** failed spinal anesthesia, incidence rate, patient position, spinal anesthesia, subarachnoid space

## Abstract

Introduction

Spinal anesthesia, a commonly used technique for lower abdominal, pelvic, and lower extremity surgeries, involves injecting a local anesthetic into the subarachnoid space to temporarily block sensory, motor, and sympathetic nerves. Despite its high success rate, the failure of spinal anesthesia, which can lead to adverse patient outcomes, remains a concern. The failure rate varies widely, from 1% to 17%, influenced by factors such as technical challenges, patient anatomy, and practitioner experience. This study aims to determine the incidence and causes of spinal anesthesia failure in a university hospital setting.

Methodology

This cross-sectional, prospective observational study was conducted at Dr. D.Y. Patil Medical College and Research Centre, Pimpri, Pune. Data were collected from patients aged ≥18 years who underwent spinal anesthesia over one year. The study recorded various parameters, including patient demographics, surgical details, and anesthetic techniques. Failed spinal anesthesia was categorized into total failure (no block achieved) and partial failure (need for additional analgesia). Statistical analysis was performed to identify the incidence and contributing factors to spinal anesthesia failure.

Results

Out of 3933 patients receiving spinal anesthesia, 72 experienced failure, resulting in an incidence rate of 1.83%. The majority of failures were total (87.5%), with partial failures accounting for 12.5%. Among the cases of failed spinal anesthesia, failures were most common in obstetric surgeries (37.5%), followed by orthopedic (25%), general (22.22%), and urologic (15.28%) surgeries. The highest failure rate according to surgery type in all patients receiving spinal anesthesia was seen in orthopedic surgery (3.46%) followed by urologic surgery (2.17%). Elective surgeries had a lower failure rate (1.39%) compared to emergency surgeries (3.87%). Quincke-type needles were predominantly used, and spinal anesthesia was most often administered at the L3-L4 intervertebral space in the sitting position. Notably, first-year residents had the highest failure rate (43.06%), while more experienced practitioners had lower failure rates.

Conclusion

The incidence of spinal anesthesia failure in our study was 1.83%, with total failures being more common than partial failures. Factors such as surgical type, emergency status, and practitioner experience can impact the failure rate. The higher failure rate among less experienced practitioners underscores the need for improved training and expertise. Regular monitoring and refinement of spinal anesthesia techniques are essential to enhance patient safety and optimize anesthetic care.

## Introduction

Spinal anesthesia (SA) is a type of central neuraxial anesthesia that involves a transient blockade of motor, sensory, and sympathetic nerves. This is accomplished by administering a local anesthetic, often combined with additional adjunctive agents, into the subarachnoid space. It is a widely utilized anesthetic technique, particularly in surgeries involving the lower abdomen, pelvis, and lower extremities [1]. Despite its broad application and generally high success rate, instances of failed SA remain a significant concern due to the potential for adverse patient outcomes.

Studies have shown that the failure rate of SA varies widely, with reported rates ranging from 1% to 17% [2]. Factors that may contribute to the failure of SA include the inability to confirm the free flow of cerebrospinal fluid, the selection of local anesthetic and adjuvants, technical challenges associated with a patient's body morphology (such as kyphoscoliosis), the judgment and experience of the anesthesiologist, and variations in CSF volume [3].

To manage an inadequate block, strategies include repositioning the patient to enhance anesthetic spread, repeating the injection, having the surgeon administer additional local anesthetic, using systemic sedation or analgesics, or opting for general anesthesia (GA) [4].

Failed SA presents several challenges including extended operating times, inadequate analgesia that may not be sufficient for the surgery, and the need for repeated attempts following a failed dural puncture. This can increase the risk of local anesthetic toxicity, and high SA, necessitate conversion to GA, and increase the incidence of post-dural puncture headache. Such conversion exposes patients to a higher risk of complications associated with GA and elevates respiratory risks [5].

Understanding the incidence and associated factors of failed SA is essential for improving clinical outcomes, refining anesthetic techniques, and minimizing patient risk. This study aimed to determine the incidence and causes of SA failure in a university hospital.

## Materials and methods

The study is a cross-sectional, prospective, and observational type of study. Following approval from the institutional ethics sub-committee (Ref no: I.E.S.C./88/2023) and obtaining informed written consent, data collection was started. The study included all patients aged 18 years and older who received SA at our hospital for a period of one year. The team of anesthetists responsible for administering SA managed their procedures independently, with the researcher having no involvement in the anesthesia management.

The main objective of the study was to determine the incidence of unsuccessful SA including both total and partial failures and to identify factors potentially associated with these outcomes. Total failure was defined as the inability to locate the subarachnoid space with lumbar puncture, the absence of motor or sensory block within 10 minutes after injecting the local anesthetic, or the necessity for repeated SA or GA. Partial failure was defined as the requirement for additional anesthetic agents such as ketamine, propofol, or opioids for pain relief during the operation.

The study recorded the following parameters: age, weight, height, body mass index (BMI), American Society of Anesthesiologists (ASA) status, type of surgery, whether it was elective or emergency, spinal anatomy, patient positioning during the spinal procedure, technique used (median or paramedian), puncture level, type of spinal needle, the practitioner's expertise level (years of experience), type of local anesthetic, addition of adjuvant drugs to the local anesthetic, loss of drug during injection, patient position post-injection, duration of surgery, assessment of motor and sensory blocks and the measures taken in cases of unsuccessful SA.

## Results

A total of 3933 patients received SA over a period of one year. Out of these, failed SA was reported in 72 patients. The incidence of failed SA was 1.83%.

Demographic information on these patients is summarized in Table 1.

**Table 1:** Demographic data of the patients with failed spinal anesthesia

	Mean ± SD	Range
Age (Years)	36.63 ± 17.34	18-82
Height (cm)	159.30 ± 7.3	145-179
Weight (Kg)	59.74 ± 12.11	35-107
Body Mass Index (Kg/m^2^)	23.51 ± 4.39	14-37.9
Duration of Surgery (min)	64.10 ± 21.33	31-126

Out of 72 patients, 59 patients belonged to ASA class I (81.94%) and 13 patients belonged to ASA class II (18.06%).

Failed SA was noted in a total of four different surgical departments. Obstetric surgeries were the most common type contributing to 37.5%. These were followed by orthopedic surgeries (25%), general surgical procedures (22.22%), and Urological surgeries (11%). However, the SA failure rate according to surgery type was highest in orthopedic surgeries (3.46%) followed by Urological surgeries (2.17%) (Table 2).

**Table 2:** Unsuccessful spinal anesthesia (SA) by type of surgical operation

Operation type	Failed SA		Total operations, n	SA failure according to surgery type, %
	n	%		
Obstetric Surgery (Cesarean Delivery)	27	37.5	1453	1.85
Orthopedic Surgery	18	25	519	3.46
General Surgery	16	22.22	1455	1.09
Urologic Surgery	11	15.28	506	2.17
Total	72	100	3933	-

A total of 62.5% of the failed spinal procedures were elective cases (n=45) while the rest 37.5% of cases were emergency cases (n=27) (Table 3).

**Table 3:** Rate of failed spinal anesthesia (SA) in elective and emergency surgeries

Operation type	Failed SA		Total operations, n	SA failure according to surgery type, %
	n	%		
Elective	45	62.5	3236	1.39
Emergency	27	37.5	697	3.87
Total	72	100	3933	-

Quincke-type needles were used for SA in all patients. The majority of these were 26-gauge needles used in 58 patients (80.55%). Twenty-five-gauge needles were utilized in seven patients (9.72%) and 23-gauge needles were used in another seven patients (9.72%).

Most of the patients with failed SA had normal spine anatomy. Three patients had scoliosis of the spine on examination (4.17%). SA was administered in a sitting position in all patients. In the majority of the patients, the spinal needle was inserted at L3-L4 intervertebral space (n=60, 83.33%) followed by L2-L3 space in 10 patients (13.88%), L1-L2 space in one patient (1.38%) and L4-L5 space in one patient (1.38%). The median approach was used for needle insertion in all patients except one. After confirming clear and free flow of cerebrospinal fluid, Inj Bupivacaine (Heavy) was administered in the subarachnoid space in all patients and no loss of drug was observed during injection. Fentanyl 25 µg was administered as an adjuvant along with the local anesthetic in 43 patients (59.72%) and clonidine 30 µg was administered as an adjuvant in two patients (2.77%). All patients were made supine after injection of the drug.

The incidence of failed SA was 1.83% out of which 87.5% of failed spinal were total failures and 12.5% were partial failures (Table 4).

**Table 4:** Rate of total and partial failures of spinal anesthesia

Type of failure		Number	Percentage
Total failures	Repeat spinal	40	55.56
	Converted to general anesthesia	23	31.94
Partial failures	Sedation	9	12.5
Total		72	100

The least experienced practitioner had less than 1 year of anesthesia experience (a first-year resident), while the most experienced had 10 years of anesthesia experience. Table 5 presents the failure rate according to the anesthetist’s years of experience (Table 5).

**Table 5:** Failure rate based on work experience of anesthetist

Years of experience	N(%) of failed spinal anesthesia
<1	31 (43.06%)
1-2	26 (36.11%)
2-3	6 (8.33%)
>3	9 (12.5%)
Total	72 (100%)

## Discussion

SA is one of the most commonly utilized anesthesia techniques in modern medical practice, particularly for surgeries involving the lower abdomen, pelvis, and lower extremities. Its popularity stems from its effectiveness, relative safety, and the rapid onset of anesthesia it provides. However, despite its widespread use and general success, the failure rate of SA is not negligible, with studies reporting a variation between 1% and 17% [2,6,7,8]. This inadequacy can involve one or more aspects of the block: its extent, quality, or duration with any combination of these factors potentially being suboptimal [4]. The rate of SA failure in our study was found to be 1.83%.

Failed SA poses several challenges, such as prolonged operating times, insufficient analgesia, and the need for multiple attempts after a failed dural puncture. This increases the risk of local anesthetic toxicity, potential high or total SA, and may require conversion to general anesthesia (GA), which carries a greater risk of complications and respiratory issues [5]. Compared to GA, SA may lower the 30-day mortality rate for patients at intermediate to high cardiac risk undergoing surgery and decrease the likelihood of postoperative pneumonia [9,10]. Sung et al., in their study, found that during cesarean sections, the GA group was associated with greater maternal blood loss and a higher percentage of newborns with 5-minute Apgar scores below 7 compared to the SA group [11]. Postoperative delirium and early cognitive dysfunction are more prevalent in elderly patients receiving GA. Additionally, elderly patients may benefit from SA, as it tends to result in fewer postoperative complications and improved recovery outcomes [12].

The most frequent instances of failure were observed in obstetric surgeries where the failure of SA was attributed to an inability to provide sufficient analgesia for the procedure. This may be due to several factors, including the technical challenges and anatomical changes resulting from the enlarged uterus and the discomfort of labor in obstetric patients. The pressure applied to the uterus during delivery often causes pain for the mother potentially necessitating sedation post-delivery [13]. However, the highest SA failure rate according to surgery type was seen in orthopedic surgery followed by urologic surgery. The high failure rate in orthopedic cases could be attributed to the difficulty of positioning patients optimally due to the pain at the fracture site. This may necessitate multiple attempts to dural puncture [14]. The calcification of bones and ligaments in elderly urologic patients has a similar impact [15].

Our anesthetists typically administer SA with the patient in a sitting position. It has been suggested that anesthetists should develop proficiency in the more challenging lateral approach, enabling them to perform either technique with confidence. A lack of expertise in the lateral position could lead to failed SA and the need to convert to GA, especially in cases of emergency cesarean delivery where patients with fetal compromise are unable to sit up safely [14,16]. In our study, 3.87% of emergency surgeries requiring SA experienced a failed SA compared to 1.39% of elective procedures. The higher incidence of failed SA in emergency surgeries aligns with findings from a study by Ashagrie et al., which reported that the likelihood of SA failure is seven times greater in emergency surgeries compared to elective ones [17].

Harten et al.’s study concludes that height and weight are important factors in predicting the final level of the block [18]. The study by Kim et al. found that both obesity and bupivacaine dosage are significant predictors of the outcomes of SA [19]. Therefore, adjusting the dose of bupivacaine according to these factors should achieve an optimal dermatomal level of anesthesia and enhance both the effectiveness and safety of SA [18,19]. Fuzier et al.’s multicenter prospective study conducted in France demonstrated that not using adjuvant medications with local anesthetics results in a twofold increase in the failure rate of SA. It has been noted that adjuvants enhance patient comfort, strengthen the effects of local anesthetics, and reduce the need for additional analgesia during surgery [7]. Adjuvant was not administered in 37.5% of failed spinal cases in our study.

As a university hospital, many procedures are carried out by residents which could account for the highest failure rate of 43.06% observed among first-year residents with less than one year of experience. As experience increased, the failure rate decreased, emphasizing the critical role of proper training and experience in minimizing the risk of SA failure. This finding aligns with previous research conducted in a Nigerian teaching hospital which also showed a strong correlation between the anesthetist’s work experience and the success of SA [15]. With increased experience, errors such as misplaced injections, incorrect solution selection, improper dose determination, inaccurate positioning, and inappropriate needle insertion during SA can be avoided. This helps prevent issues like unilateral or inadequate sensory height in SA [20].

Our study has certain limitations. Failed SA procedures were not followed up post-surgery and postoperative complications were not evaluated. Additionally, we did not assess the relationship between failure rates and patient satisfaction. Better comparisons could have been achieved if parameters were recorded for all patients receiving SA, rather than limiting the data collection to only those with failed SA. Demographic factors and patient populations vary widely across different regions, so these findings may not be universally applicable.

Service quality, performance metrics, and patient safety are becoming increasingly important and consistently monitored in healthcare settings [21]. As these factors directly impact patient outcomes and overall satisfaction, they necessitate ongoing evaluation and improvement efforts. In this context, the success rates of SA should be regularly assessed to ensure that the procedure meets the highest standards of care. Continuous monitoring allows for the identification of trends or issues that may affect the effectiveness of SA, providing opportunities to refine techniques, update protocols, and implement best practices. By striving to improve the quality of the procedure, healthcare providers can enhance patient safety, reduce the incidence of complications, and ultimately improve the overall effectiveness of anesthetic care.

## Conclusions

The incidence of failed SA in our study was 1.83%, with the highest failure rate observed in orthopedic surgeries, likely due to positioning challenges and patient factors. Emergency surgeries had a significantly higher failure rate than elective ones, highlighting procedural challenges in urgent scenarios. Furthermore, failure rates decreased with increased anesthetist experience, underscoring the importance of enhanced training, tailored protocols, and procedural refinements to improve SA success and patient outcomes.
